# Recovery post treatment: plans, barriers and motivators

**DOI:** 10.1186/1747-597X-8-6

**Published:** 2013-01-30

**Authors:** Paul Duffy, Helen Baldwin

**Affiliations:** 1 Criminal Justice Manager, Centre for Public Health, Liverpool John Moores University, 2nd Floor, Henry Cotton Campus, 15-21 Webster Street, Liverpool, L3 2ET, UK; 2 Public Health Researcher/Analyst, Centre for Public Health, Liverpool John Moores University, 2nd Floor, Henry Cotton Campus, 15-21 Webster Street, Liverpool, L3 2ET, UK

**Keywords:** Recovery capital, Treatment, Abstinence, Employment, Family, Motivators, Peer support, Accommodation, Drugs, Alcohol

## Abstract

**Background:**

The increasing focus on achieving a sustained recovery from substance use brings with it a need to better understand the factors (recovery capital) that contribute to recovery following treatment. This work examined the factors those in recovery perceive to be barriers to (lack of capital) or facilitators of (presence of capital) sustained recovery post treatment.

**Methods:**

A purposive sample of 45 participants was recruited from 11 drug treatment services in northern England. Semi-structured qualitative interviews lasting between 30 and 90 minutes were conducted one to three months after participants completed treatment. Interviews examined key themes identified through previous literature but focused on allowing participants to explore their unique recovery journey. Interviews were transcribed and analysed thematically using a combination of deductive and inductive approaches.

**Results:**

Participants generally reported high levels of confidence in maintaining their recovery with most planning to remain abstinent. There were indications of high levels of recovery capital. Aftercare engagement was high, often through self referral, with non substance use related activity felt to be particularly positive. Supported housing was critical and concerns were raised about the ability to afford to live independently with financial stability and welfare availability a key concern in general. Employment, often in the substance use treatment field, was a desire. However, it was a long term goal, with substantial risks associated with pursuing this too early. Positive social support was almost exclusively from within the recovery community although the re-building of relationships with family (children in particular) was a key motivator post treatment.

**Conclusions:**

Addressing internal factors and underlying issues i.e. ‘human capital’, provided confidence for continued recovery whilst motivators focused on external factors such as family and maintaining aspects of a ‘normal’ life i.e. ‘social and physical capital’. Competing recovery goals and activities can leave people feeling under pressure and at risk of taking on or being pushed to do too much too soon. The breadth of re-integration and future plans at this stage is limited primarily to the recovery community and treatment sector. Services and commissioners should ensure that this does not become a limiting factor in individuals’ long term recovery journeys.

## Background

### Recovery agenda

In recent years there has been a fundamental shift toward recovery-oriented models of substance misuse treatment, with policies and practices becoming increasingly focused on achieving sustained recovery from substance misuse [1-3]. Previous UK drug strategies have aimed to increase participation in treatment and improve retention rates [4,5], whereas the current strategy intends to move beyond harm reduction to, “offer every support for people to choose recovery as an achievable way out of dependency” [6].

### Defining recovery

There are concerns that this recovery movement is ahead of the development of an evidence base for its implementation [7-9]. A lack of clarity and agreement about the meaning of recovery has prompted debate about the goals of treatment and created difficulties for drug treatment commissioners and practitioners [7,10]. However, in general terms recovery is thought to be characterised by voluntarily sustained control over substance use, health and wellbeing, and participation in society [10,11].

### Recovery capital

An individual’s ability to recover from substance misuse can be understood in terms of their ‘recovery capital’; the resources they can draw upon in the initiation and maintenance of recovery [12]. Resources may stem from their social networks, education, employment, financial assets, health, beliefs and values etc. Recovery capital can also be considered a way to conceptualise the barriers to and facilitators of recovery with higher levels predicting sustained recovery from substance misuse [13] and negative recovery capital, such as mental illness or incarceration, impeding one’s capacity to recover [12].

While total abstinence may not be a pre-requisite for recovery, findings suggest that most ‘self-remitters’ – people whose recovery capital enables them to overcome addiction unaided by treatment – choose to abstain from future substance use [14]. Recovery capital is also thought to be accumulated over time as a person remains abstinent from drugs and alcohol [15,16].

There is a substantial body of literature, mostly from the USA, much of which examines the predictive value of aspects of in-treatment recovery capital and the usefulness of interventions aiming to boost aspects of recovery capital for longer term outcomes such as abstinence and preventing re-admission to treatment. Supportive relationships with peers, families and communities are suggested to be critical for ongoing recovery from substance misuse [17-21]. Peer support has been associated with intention to change substance use, improved functioning, self-efficacy and quality of life [22-24]. Peer-based mutual aid groups have become increasingly popular internationally; Alcoholics Anonymous (AA) and Narcotics Anonymous (NA) are now widespread across the UK [2,25,26]. Positive family relationships have been found to reduce the likelihood of relapse to substance use [27,28], although re-building relationships with family members and restoring trust can be a difficult process [29]. In light of this evidence the UK Government has a stated aim to support communities to build recovery networks involving peers, families and carers [6].

Employment increases legitimate income and can improve living standards, both of which are important for recovery [12,30]. However recovering substance users face a range of barriers to employment, including a lack of qualifications and experience, low self-esteem, health problems, and criminal records [31,32]. Studies highlight a need for joint working between drug treatment commissioners, drug treatment services, employment services and employers to help substance users find work [33-36]. For example, delivering vocational training services alongside treatment programmes has proven to be effective [32,37-39]. Furthermore, interventions to address poor spending habits among people with substance use problems have achieved positive outcomes, both in terms of money management and substance use [40-42].

Substance misuse can increase the risk of homelessness [43,44] and in turn, residential instability can increase substance use and lead to treatment re-admission [43,45], although it seems the relationship between homelessness and substance misuse is a complex one [44]. Addressing housing needs can produce positive treatment outcomes [46] and supported housing for people in recovery has been associated with reduced substance use, fewer arrests and increased likelihood of obtaining permanent housing and employment [47-50].

Co-morbidity of mental health and substance use issues is common internationally and is associated with increased risks of relapse, suicide and incarceration [51-53]. Therefore the importance of mental health for recovery needs to be recognised. Poor physical health may impede efforts to recover from substance misuse [12] and ill-health at treatment entry which is not addressed has been shown to predict later poor physical health and mortality post treatment [54,55]. Substance misuse treatment that addresses individual mental and physical health needs can have positive health outcomes and reduce substance misuse [56-59]. Furthermore, treatment clients have expressed interest in interventions designed to improve their health [60-62].

Aftercare is a critical part of building recovery capital. Engagement in self-help groups and meaningful activities can help build peer networks and provide structure to the lives of those in recovery [22,63,64]. Aftercare services have been shown to reduce substance use, delay relapse, lower stress and improve quality of life [65-68]. The current UK drug strategy urges treatment providers to form close links with aftercare services, with the aim of building a recovery focused treatment system which delivers ‘end to end’ support [6].

### Research aims

Additional evidence (especially from the UK) is required examining the factors that those in recovery perceive contribute to or impede sustained recovery from substance misuse [13,22]. Previous research has tended to focus on quantitative investigations of recovery initiation and short-term treatment outcomes such as the achievement of abstinence. People continue to face many challenges long after gaining control over their substance use, particularly in relation to employment, housing and relationships [69].

This research aimed to identify the factors (recovery capital) that play a role in recovery post treatment by using a qualitative approach to examine the views and experiences of individuals who have recently completed treatment in a wide variety of settings. Findings will add a British perspective to the relatively small amount of work on post treatment recovery capital, help guide the development and delivery of recovery-oriented models of treatment and examine the role that policy can and does play in sustained recovery.

## Methods

This research adopted a generic qualitative approach which drew on grounded theory methodology. This is an approach commonly used in applied health research (the authors’ area of expertise) which seeks to understand people’s experiences and perceptions in relation to a particular clinical problem or process, without conforming to any one traditional methodology [70,71]. This approach was considered to be the most suitable for this work due to it being exploratory in nature, attempting to generate theory around the relatively new recovery capital concept within an under researched population (clients having left treatment), similar to grounded theory [72,73], while relating participants’ experiences (drawing on phenomenological approaches [74]) to existing literature on recovery capital and developing guidance for future policy and practice. A quantitative design was determined to be inappropriate given the aim of the study was to examine individuals’ perceptions across a number of areas, some of which were potentially unknown to the researchers, and not to quantify levels of recovery capital. Provisions were made to ensure the trustworthiness of the work in accordance with relevant guidance from the literature in relation to the recruitment of participants and collection and analysis of data [75,76], as detailed in the following sections. Furthermore, researchers sought input from peers external to the study on the research design and process.

### Recruitment

Participants were a purposive sample; clients completing treatment successfully and not continuing in structured treatment were recruited in line with the study’s aim to examine recovery post-treatment. Participants were recruited opportunistically following exit from one of 11 drug and alcohol treatment agencies in a predominantly urban area in the North of England, characterised by relatively high levels of deprivation and long standing substance use problems. The agencies included two residential rehabilitation services (one Therapeutic Community and one 12 step focused), a community rehabilitation service, one community aftercare service, one criminal justice service and six community drugs teams. Agencies were selected to cover a broad spectrum of treatment approaches and therefore a diverse participant group. Sessions were undertaken with key workers in each service to outline the aims of the research, seek their assistance with recruitment and receive feedback on suggested interview topics. Clients reaching the end of their (successful) engagement with the service, who were not being referred on to further structured treatment, were asked by their key worker to participate in the research and were given written and oral explanations of their involvement. Clients were given the opportunity to refuse to participate or withdraw at a later date. If they agreed to participate, a consent form was signed and provided to the research team with contact details for the clients. Researchers made contact with the clients once they had left treatment and interviewed them between one and three months later. A ten pound shopping voucher was provided to recompense participants’ time.

### Participants

The 45 participants (18 from residential services) were predominantly male (n = 30) and White British (n = 43) with ages ranging from 22 to 54 (mean = 39, s.d. = 7.77). The substances that people had sought treatment for were varied with most (n = 40) indicating poly-substance use (most often opiates with cocaine and/or crack). Five participants had sought treatment for alcohol use only. At the stage that recruitment took place, all participants were engaged in treatment voluntarily however mandated treatment had made up a part of five participants’ treatment journeys.

### Data collection

Data were collected through semi-structured qualitative interviews conducted by two researchers (the authors) experienced in interviewing this participant group. An extensive literature review was conducted to identify the potential overarching topic areas (historical drug use and treatment experience, relationships, offending, future plans, accommodation, activity, employment and motivation). These topics, and the open questions formed as prompts from them, were reviewed by practitioners in each site and two researchers from the field not directly involved in the project. These formed the basis for the interview schedule but interviewees were encouraged to freely discuss their own experiences of recovery whilst the researchers ensured all areas were covered in every interview as a minimum. An example of the opening question for a topic area would be *‘How confident do you feel about continuing to tackle your problems with drugs and/or alcohol? Why?’* Researchers were able to adapt to responses and incorporate additional prompts and direction into subsequent interviews. Interviews were conducted in private, usually in participants’ homes, with a view to facilitating open and honest reflection. All interviews were recorded and transcribed and field notes were made by the researchers. Recruitment was continued until interviewers noted saturation i.e. few new themes emerging with the repetition of themes identified in previous interviews and preliminary analysis [77].

### Analysis

Interview transcripts were analysed by the researchers who had conducted the interviews. The researchers used a general inductive approach to thematic analysis which incorporated both inductive and deductive coding [78,79]. This analytical approach was appropriate for this study as the authors aimed to generate an understanding of participants’ experiences that was not limited by a pre-determined hypothesis, while also identifying themes that would relate to previously identified domains of recovery capital.

Data analysis was performed using NVivo 9 software [80]. Overarching topics identified through the literature review formed general categories for the analysis (relationships, future plans, employment etc.). Inductive coding of the data then led to the creation of themes which were either arranged beneath these general categories or combined to form new categories. Measures were taken to ensure the rigour of the analysis conducted in line with guidance on qualitative data analysis. For example, researchers coded the data independently and maintained an audit trail of their procedures, interpretations and coding decisions before discussing their emergent themes, and where there was discord in final themes identified, coding was revisited jointly to reach consensus [76,77]. Data analysis was also an on-going process which involved comparisons between new and existing themes and categories and refinement of concepts and associations through further data collection and analysis [73,77].

### Ethics

The research was approved by the university’s research ethics committee. Written informed consent was obtained from each participant, initially by key workers to allow contact by the research team and then by the research team prior to interview.

## Results

### Participants’ backgrounds

Most interviewees (especially opiate users) had been in treatment previously. Those engaged due to alcohol or cocaine use were less likely to have had multiple periods of treatment. Previous treatment compliance was commonly described as partial with non-adherence to prescribed medication, a lack of commitment to sessions in residential treatment and a lack of motivation to become abstinent. Opiate and crack users (OCU) reported extensive offending histories including numerous prison stays whilst those reporting alcohol use or drug use which did not include opiates or crack (ALCNON) indicated less extensive offending histories if any.

All participants viewed their most recent treatment episode as a successful one, although the content and nature of the treatment was not universally felt to be positive. Reasons why this particular episode of treatment was viewed as a success primarily revolved around participant factors rather than treatment delivery. A focus on internal factors rather than external was central, with an increased control over emotions and ‘opening up’ highlighted. Counselling and peer support groups were implicated as a mechanism for this in a number of cases. Improved cognitive processes were also indicated as participants felt they had an increased understanding of addiction and had come to a realisation that drug use was a choice. The timing of interventions was critical with a perception that the same treatment or support provided at a different time would not have proved successful.

‘It’s about fixing your inside. Getting to grips with who you are, how you are, being comfortable and then adding these things to your life.’ Male, OCU, 37

Whilst most interviewees had remained completely abstinent (from all substances) since completing treatment, lapses had occurred for some clients and a small number saw no problem with continuing some substance use (generally not of the substances they had viewed as problematic) because they felt they had established a better level of control.

### Aftercare and engagement in post treatment activity

Most participants had engaged in aftercare. In some cases this was organised by their treatment agency before discharge but there were a lot of self-initiated contacts with the perception being that aftercare is available but you have to look for it.

‘You’ve got to be pro-active most definitely, you’ve got the resources available to you.’ Male, ALCNON, 38

Participants exiting residential treatments were more likely to cite their treatment agency as being central to arranging aftercare. In contrast people leaving community treatment were more likely to report arranging their own aftercare or indicate that they did not want or need ongoing support.

‘No, no, no, I didn’t need it and I didn’t ask for any help, because as I’ve said, they did offer support, you know, on my discharge appointment but I said I didn’t need it.’ Female, ALCNON, 36

In some cases people felt support waned after the initial stages of aftercare. Whilst most participants didn’t feel this had been a major issue there was a feeling it might be a problem for less motivated treatment leavers.

The generally motivated nature of this group of participants is suggested by their perception that they were finding it easy to fill their time, something that was felt to be critical for their continued recovery. A ‘proactive’ philosophy among this group was also reflected in reports of playing a role in engaging other treatment leavers into aftercare. Participants who were struggling to engage in activities cited their own lack of motivation rather than a lack of availability.

‘I know for a fact it’s dangerous for me to sit around doing nothing because if I do me head will have me off. I’m 110% sure of that, I need something to do I need structure. Even if it’s just getting up and you know doing something instead of just sitting around all day that’s when it will come and bite me again I know it will.’ Male, OCU, 39

A wide variety of aftercare was considered beneficial, often focused on non substance use related outdoors activity or creativity (drama, dance, creative writing, music). Therapeutic aftercare was also engaged in including the 12 step mutual aid programmes (NA/AA/CA) attended by a large number of participants. These were felt to be essential by participants regardless of the philosophy of their original treatment, so essential, in some interviewees’ opinions, that missing a meeting would lead to relapse.

‘I maintain me recovery with Narcotics Anonymous now. That’s how I keep clean today. I don’t go without going once a week. Just I go there and remind you see, with addiction it’s, disease tells me I haven’t got a disease, tells me I’m alright, I’m OK now which is complacency and that’s when your disease sneaks up on ya bites you on the arse.’ Male, OCU, 37

Attention was drawn to the competing priorities that could arise when engaged in a number of recovery activities with different agencies. This had led to some people reducing or dropping attendance at potentially important activities helping to prevent relapse.

‘I wasn’t getting to enough meetings recently I had too much on ‘cause I’ve that college course that I did.’ Female, OCU, 32

### Social support

Social isolation was a particularly strong theme to emerge with reference to participants’ unintentional or enforced separation from positive relationships. For drug users within the sample this was often framed within the repetition of a lifestyle that becomes solely about the process of obtaining drugs, leaving no room for anyone or anything else. Despite this historical isolation, participants, with few exceptions, felt that they now had sufficient social support, almost exclusively from others in recovery. There were few examples of established relationships outside recovery. The value placed on peer support throughout the recovery process (during and after structured treatment) was obvious and the positive reinforcement of seeing others in recovery was highlighted as critical. This was evident in feedback regarding 12 step mutual aid groups where the primary reason for attendance was a sense of ‘belonging’ or being able to relate due to shared experiences.

‘When you sit there you hear stories of people, what they were going through, in the madness, when they were using, drinking or drugs like. You relate to them, a lot. It reminds you of where you were and that place you don’t ever want to go back to. That’s what I get out of my meetings.’ Male, OCU, 42

The concept of ‘giving something back’ was evident throughout interviews. Participants were often engaging in drugs related voluntary activities (work visiting schools was often indicated), were already or intended to get involved in formal peer support by completing peer mentoring courses or had an eventual goal of working within the drug and alcohol treatment sector.

‘I’ve been doing some voluntary work as a recovery mentor as well. That’s been good because you get a lot of good feedback from peers up there.’ Male, OCU, 35

The dissolution of family or other close relationships due to substance use was a common theme although this was less often the case for ALCNON participants who reported maintaining at least some relationships with close family members. Although separation from family was often self-imposed, an integral part of participants’ recovery was the re-establishment or improvement of these relationships, especially with children. Indeed family contact was the key motivator stated by participants for maintaining their recovery.

‘Now that I’m off everything, they all seem to be back and they’re there for me again so it, you know, it’s a good thing really.’ OCU, Female, 34

Despite the importance of re-establishing these relationships, direct family involvement in treatment was rare. This may be linked to participants’ feelings that they still had some way to go to convince family members despite their positive progress. This lack of trust was also evident in difficulties that were being experienced by some clients in obtaining access to their children.

‘Well to tell you the truth I’ve not been allowed to contact them, their mother put stumbling blocks every step of the way, she once said to me go to rehab get clean and you know hundred per cent access and you know she fucking flipped it on the head you know what I mean.’ OCU, Male, 44

### Accommodation

Stable, appropriate accommodation was the norm for most participants, linked to the high number of individuals who were in supported housing at the time of their interview. The importance of supported housing was one of the themes to emerge most strongly and there was a feeling that there was good availability within the area. Critical aspects of supported housing were the proximity of peer support, abstinence checks and the staged re-introduction to independent living.

Interviewees were split on whether they wanted to move towards more independent living with a predominant feeling among those in supported housing that they wanted to stay there for as long as they could, citing the risk of relapse if they moved to independent living too quickly. Those wishing to leave supported housing were often doing so to provide more appropriate accommodation for family and relationship building but finding suitable property was an issue.

‘I saw me son on me own at the weekend, my ex partner said now that if I did get a flat, that he could come stay with me. So that’s the next thing, I need to consider. Because obviously he can’t come and stay here [in supported housing] for millions of reasons and I understand that, but I’m, I’m going to start thinking about the time to get a flat.’ Male, ALCNON, 51

The link between finances and housing was at the forefront of participants’ thinking. The inability to pay for current or future housing without benefits was highlighted both by those in supported housing and those living independently.

‘You see that’s another thing really I can’t get a job, if I wanna stay here I can’t work for two years because they get funding or something through the housing benefit for my support.’ Female, ALCNON, 44

### Employment and finances

Whilst around half of the sample had previously worked this was most often in unskilled roles, was a substantial period of time ago and jobs had eventually been lost due to drug use. The desire to return to work was strong among participants and for the most part this was accompanied by a clear vision of a pathway to employment.

‘I’ve got a few courses underneath me belt and things like that, but I’d like a cleaner business. Like to go self employed and own a cleaning business.’ Male, OCU, 42

Work with young people was indicated as one possible employment field but by far the most commonly desired field of work, particularly among previous OCU, was within drug and alcohol treatment with participants feeling they had valuable experiences to impart, a view which would appear to have been reinforced or prompted by key workers.

‘It’s just in the alcohol and drug field that’s where I want to go on ‘cause I know so much and that’s all I know really so why not put it to good (laughs) yea.’ Male, OCU, 39

Despite the desire to work, only three participants reported being employed at the time of their interview (two of which were in substance use related services) and there was a general feeling that getting back into paid employment would not and should not be rushed. There were perceived to be relapse risks associated with taking a paid job or the wrong job. Any steps being taken towards employment (aside from the three individuals in employment), were at this stage and for the foreseeable future, following the voluntary route or involved education or training. Participants were particularly conscious of their lack of basic skills, with Maths, English and computer literacy courses planned or already engaged in.

‘Early in recovery I think it can be quite dangerous to go into work straight away, I’ve seen a few people go into work straight from treatment thinking they’re ok and they’ve relapsed within a month because their first pay check’s come in.’ Male, ALCNON, 29

Perceived practical barriers to employment were fairly few although this in part may be due to the preponderance of plans to work in the treatment sector where participants’ pasts are more likely to be accepted. Issues highlighted included a lack of qualifications, poor health and a fear about the impact of employment on benefits.

Participants were generally relying on welfare payments for income (most often Employment Support Allowance (ESA), support for those who are not in a position to work due to health issues) and there was anxiety expressed about the removal of ESA as they continued in recovery and the ‘tightness’ of finances.

‘If you can pick a pen up yeah you’re fit for work. A few people who have been for the medical in this place have been thrown off it (ESA).’ Male, ALCNON, 33

Despite this identified anxiety the need for ongoing support with finances was not universal with as many participants feeling they had the skills to manage their money as those that felt they needed additional support.

### Health

A variety of long term physical health conditions were described by participants, in particular Hepatitis C (not treated in some cases), respiratory problems and circulatory issues, as well as liver, kidney and pancreatic abnormalities. Access to appropriate health care was generally good. Additional support in this area was not a priority, reflecting participants’ positive perceptions of improving health, increasing exercise and weight gain. Ongoing health issues were not universal with around half of participants indicating that they had no long term conditions, describing themselves as ‘lucky’.

‘I’m lucky in that respect, no I am, I feel very well mentally and physically.’ Female, ALCNON, 44

Historical mental health issues were common including suicidal ideation but strong improvements in this area were a feature of participants’ recovery with a feeling that drug use had been the primary cause of mental health issues. In contrast where an ongoing need for support was identified it was generally allied to a perception that drug use had been used to mask underlying issues.

### Smoking

Despite the bulk of participants indicating they were completely abstinent from drugs and alcohol there was a large proportion still smoking (addicted to nicotine). Whilst some participants expressed a desire to quit an equal number saw this as a less harmful addiction which they were not prepared to tackle at this time (sometimes stated as being on the recommendation of previous key workers).

### Confidence, motivators and barriers to continued recovery

Generally participants were confident in their ability to maintain their recovery (whether this involved complete abstinence or not) but there was an awareness that doing so required hard work.

The reasons behind this confidence generally focused on internal processes including a faith in their own determination and skills, a better understanding of addiction (particularly for those that had been through residential interventions), a realisation that drug use is a choice, a belief that the key issues had been dealt with, a general contentment with life and an acceptance that they will think about using or can see others use without triggering their own use. Underpinning most responses was the feeling that they had ‘just had enough’.

‘I think you come to the point where you just totally, you’re ill and you’re totally sick of it.’ Male, ALCNON, 46

Motivators for continued recovery revolved around two key factors. Firstly, the impact of addiction on family members (often children), including a sense of ‘not being there’, damage done and the collapse of relationships. Secondly consciousness of what had been lost or could be lost if they returned to substance use or old behaviour patterns.

‘I’ve got everything. I’ve got me kids, I’ve got me family, you understand, I’ve got my partner, I’ve got her family, you know what I mean, I’ve got a network of mates. I’d lose everything this time.’ Male, OCU, 42

Despite this confidence, identified risks to prolonged recovery were numerous with six points emerging most strongly. Four were linked to relapse; social interactions or situations, ‘getting too far ahead of yourself’, having too much unoccupied time and points of high emotion or stress. Two practical barriers; finances and criminal records, were also identified.

‘Even though I’ve taken on all this stuff and a lot of it was my idea to do this, I do kind of sometimes get to the point where I think to myself, what have I taken on here? I kind of do worry about it sometimes, that I can’t fulfil all the obligations that I’ve set, but also on the other hand I can’t turn it round and do nothing you know? I have got to do something.’ Male, OCU, 34

‘Whether I get there because of me previous convictions is a different thing, you know I’ve got to be mindful of the fact that I’ve been to prison for violence and I’ve been to prison for serious robberies, aggravated burglaries and drugs, so it’s kinda like I’ve got to pick and choose how and where I work.’ Male, OCU, 34

Central to the recovery process and interviewees’ perceived ability to make progress was an increased self-awareness, the ability to manage their own emotions and communicate effectively as a result.

‘I started learning how to communicate without going, ahhh! I learnt to slow down my thought processes and to relay back and to reflect and look at my behaviour ‘cause it wasn’t all about me anymore, it was about the way I was affecting others.’ Female, OCU, 35

## Discussion

Findings have provided insight into the relative importance of aspects of ‘recovery capital’ for individuals post treatment as well as the competing pressures and challenges faced. There is little qualitative work, especially with British populations, examining recovery capital. International evidence has generally been quantitative in nature [18-20,27,30,40,42,45,48-50,56-58] and has looked at the predictive value of in-treatment recovery capital or interventions aiming to increase capital for longer term outcomes such as preventing treatment re-admission or abstinence. This work confirms in general terms the relevance, post treatment, of domains that have been identified in previous work such as good social support [18-20,27,28], secure accommodation [45,48-50], little need for additional support around mental health [52,53,56], improving physical health [57-59,69] and good financial management [40,42]. However it is the relative importance of these factors at this stage which is of interest, with certain factors appearing to have been sufficiently dealt with during the treatment process (acute physical and mental health concerns), some not appearing to register at all (financial management) and others considered important but not imminently (employment). The strongest indications were for the importance of rebuilding a social support network (family and friends) and having secure accommodation.

Participants’ improvement in their internal states i.e. ‘human capital’ which had been central to the success of their latest treatment episode was driving their ongoing confidence [9] whilst motivators were often the attainment of external ‘social’ and ‘physical capital’ e.g. family, potential loss of items associated with ‘normal’ life. As Granfield & Cloud [14] indicate abstinence is not universally a long term desire but it is for the majority and for much of this sample their future employment and relationship development could only be facilitated through ongoing abstinence [9].

The considerable contribution of peer support confirms the importance of this identified in previous work [19,20]. The ‘social contagion’ concept extended by Best and Laudet [17] to drug and alcohol recovery is evident, with the reinforcing aspect of mutual aid emphasised strongly. The perceived risk of relapse as a result of not attending mutual aid meetings does raise some questions as to the level of ‘dependence’ on such meetings. Whilst it should not be suggested that mutual aid is harmful, further work considering its potentially restricting impact on long term broadening of recovery and social interaction would be warranted. Reports of friendships outside the ‘recovery community’ were rare and this focus on interaction only with those in recovery may be necessary at this stage. More varied relationships may develop over time when mainstream training, education or employment is sought but it does raise questions as to the scope of people’s recovery. This narrow scope may also be reflected in the strong focus on future employment within the treatment sector.

Despite evidence from previous work suggesting that family involvement in treatment is critical in recovery [27-29,81] findings from this study would suggest that, for this group of successful treatment completers in recovery, it was often not an integral part of the treatment process. This did not appear to be due to a lack of opportunity to do so but a lack of desire from participants themselves or their families to pursue this. Re-establishment of these relationships would appear to be important post treatment but before then it may be difficult to overcome the ingrained lack of trust [29]. Some detailed thought might be needed on the timing of family focused interventions and their ongoing input post structured treatment. Certainly the prospect of improved family relationships in the future is a potentially strong in-treatment motivational tool.

Agencies’ roles in organising aftercare appear to be inconsistent and post treatment arrangements were often instigated by this generally motivated group of participants themselves, who shared information about potential activities with peers. Competing conclusions can be drawn from these findings. Firstly it would appear that the UK Drug Strategy’s suggestion that treatment agencies need to forge stronger links with aftercare [6] has some way to go, but secondly, that peer supplied information may be as effective as work that agencies can do to ensure treatment leavers are fully informed. The potential for peer in-reach to agencies from the large number of services providing ongoing recovery support may need further development. Logically this function could be co-ordinated by the numerous recovery champions now being formally identified across England [82].

Emphasis is placed in the most recent UK Drug Strategy [6] on helping people engage in training and employment, to reintegrate into mainstream society, and then in the more hard-line ‘Putting Full Recovery First’ paper [83] this is strengthened with references to ‘contribution’ to society. However, this work has indicated that many valued recovery activities are more about personal well being and engaging in opportunities for learning that may not lead in the short term to a direct societal contribution. None the less they are of obvious value to participants. In addition, findings suggest a potentially negative situation where structured educational or voluntary employment activities have put pressure on attendance at substance specific activities such as mutual aid which were essential for many participants in preventing relapse. This reflects a battle taking place between two stated relapse risks; doing too much and doing too little.

The role of supported housing would appear to be of great importance and while its availability was good within this geography this may not be the case in all areas. The UK Drug Strategy recognises the importance of appropriate accommodation but focuses on acute need around homelessness rather than the broader need identified here [6]. Those in recovery consider supported housing to be critical and generally desire to remain there for as long as possible. As such it should be a commissioning priority, one that will require cross sector co-operation to make the most of resources in an area that could be hit hard by continued cost savings.

Participants revealed a complex inter-relationship between paid work, accommodation, finances and sustained recovery. Those in recovery want to work and through the Welfare Reform Act, 2012 [84] the UK Government is taking steps to move people from Employment Support Allowance (welfare support for individuals who are deemed less able to work or look for work) into more active job seeking welfare streams. However, long term supported accommodation is seen as crucial for many in recovery, and this is paid for through housing benefit, which they assume could be reduced or removed due to a change in employment status. There is a fear about the ability to afford to live independently i.e. without benefits [31]. Whilst employment might solve this it was felt to bring a risk of relapse if entered into too early [35,85] and also a loss of the critical day to day peer support offered in supported accommodation. Essentially recovering drug users perceive there to be an ‘unemployment trap’ highlighted in previous work [86-89]. As such they do not seek anything other than voluntary work for a period of up to two years, a valuable stepping stone to paid employment [34]. In concordance with this, previous research would suggest that the benefits of vocational interventions delivered as part of treatment are mostly seen 12 months or more after treatment discharge [56].

A complex set of cost-benefit analyses are being undertaken that are poorly assisted by policy produced in departmental silos (particularly around welfare provision). Where individuals have worked in the past this has generally been in low skilled jobs providing minimal transferable skills. Many participants indicated relatively early onset of problematic drug use, substantially inhibiting ‘normal’ skill acquisition and it is as though they are re-starting in their late teens with few formal educational, vocational and often social skills. The time and support afforded to them around employment should be at least commensurate with that of young adults i.e. education and training could be their primary focus for at least two years and all systems should be organised to allow for this. This would reflect the view that recovery capital, in this case aspects of ‘human capital’, is accrued over time rather than being a fixed concept [15,16].

Further research should extend the time period post treatment release for speaking to clients. Whilst numerous pressures were being experienced by participants shortly after leaving treatment, feedback suggested another potential stress point 12–24 months post discharge. At this point supported housing is often no longer available, pressure to be in employment and not claiming benefits is intensified and peer support may have dissipated as recovery takes people in different directions. This, in some ways, is the true point of reintegration into mainstream society.

For many participants their plan on eventually returning to paid employment was to assist others by working in the treatment field. This is laudable and offers a route into paid employment via voluntary work that avoids a number of barriers that would be in place in other employment fields. However, this cannot be a successful long term policy direction. The numbers of jobs for treatment workers are limited and the greater the success of treatment the smaller the number of jobs, so this trend will be self-defeating. People’s desire to care should be harnessed in other forums. Further work should examine whether the lack of breadth in ambition relates to confidence in working in other arenas, doubts as to their own ability to acquire new skills (the motivation levels among this study’s participants for new learning were very high) or treatment services being limited in their advice to clients.

In the period shortly after treatment exit, few additional health concerns were emerging that required intervention not already put in place during structured treatment. Whilst health issues appeared to be further down the list of priorities for participants the role of smoking in long term health warrants further consideration. It is unclear why smoking was viewed as a separate issue to the use of other alcohol or other drugs especially as so many of the sample were vehement about their need to remain completely abstinent. This avoidance of smoking cessation may be due to lower perceived acute risks from smoking [90], a reticence among drug and alcohol treatment practitioners to implement smoking cessation programmes [91] or a lack of knowledge regarding the positive overall treatment impacts of smoking cessation [61,92-94]. A more robust approach to tackling nicotine addiction may be warranted to take advantage of participants’ generally high levels of motivation.

### Study limitations

This study specifically aimed to examine the views of self-selecting individuals who had been discharged from treatment with a successful outcome. As such they are likely to represent a group who have developed strong recovery capital and their views will be positively biased as a result of this. In addition, as treatment workers played a key role in facilitating recruitment there is the potential for them to have introduced some bias (only picking positive clients). Another source of potential bias within our sample is that we do not know how many individuals refused to take part, meaning clients with less positive outlooks may not have participated. However as the purpose of this work was not to determine the relative perceptions of successful and unsuccessful treatment completers or to look at perceptions of treatment quality but to look at ongoing challenges among a group that had successfully completed treatment these biases are not critical flaws. It should also be noted that certain findings, particularly those around accommodation availability and aftercare provision will be determined in part by the structures in place within this geography. In other areas resource availability may be different and this would be reflected in the views of those in recovery regarding adequacy of on-going support and their related confidence.

## Conclusion

The motivation, confidence and enthusiasm of those in recovery were evident among participants in this study. It is critical that this is harnessed and that barriers around competing local and national policy and fiscal priorities do not de-rail progress. Individuals in recovery need to be provide with and informed about a sufficiently broad range of opportunities to allow them to fully embrace a life outside substance use at the most appropriate pace for them.

## Competing interests

The authors declare that they have no competing interests.

## Authors’ contributions

PD conceived of the study, led on its design, conducted analysis and produced the final manuscript. HB contributed to the design and co-ordination of the study (including fieldwork), conducted analysis and completed the literature review. Both authors read and approved the final manuscript.
